# The New Antiseptics of Dr. Credé: Silver and the Silver Salts, and Their Use in Dentistry

**Published:** 1897-08

**Authors:** M. Hille

**Affiliations:** Dentist, Dresden


### Abstracts and Translations.

### THE NEW ANTISEPTICS OF DR. CREDÉ: SILVER AND THE SILVER SALTS, AND THEIR USE IN DENTISTRY.[1]

BY M. HILLE, DENTIST, DRESDEN.

Though modern surgery has turned from antisepsis to asepsis, the former is by no means superfluous. Asepsis cannot be obtained in many conditions in which the external circumstances are such

[1] Read before the Dental Association of the Kingdom of Saxony, October 24, 1896. Abstracted from the Deutsche Monatsschrift für Zahnheilkunde, May, 1897.

that microbic infection cannot be prevented, or where it has already occurred. Moreover, antisepsis often gives us quicker and better results than does asepsis. Especially is this the case in the mouth, the field of our special labors, which is a perfect culture-oven of exogenous and endogenous micro-organisms, and where aseptic procedures are entirely inapplicable. We dentists are compelled to rely on antiseptics; and if I propose a new one to you, and recommend it most warmly, it is because of the good results that I have obtained with it in various departments of our specialty.

The ideal antiseptic must possess the following properties: It must be harmless, non-poisonous, and non-irritating; it must be fatal to all pathogenic spores and microbes; it must have no deleterious or destructive effect upon the tissues; it must be in a form that renders its application possible in the most difficult localities; and, finally, it should be sufficiently far-reaching in its effects to penetrate the deeper tissues and destroy the germs that may have penetrated to them. None of our previous antiseptics fulfil these conditions. Credé believes that two new ones, the citrate of silver and the lactate of silver, really do so; and his conclusions are confirmed by those of Halsted, Beyer, and others. His bacteriological and clinical experimentation in the Carola Hospital, of Dresden, have given most surprisingly good results.

We dentists are well aware of the fact that the precious metals, in proper form, hinder the growth of the schizomycetes; that gold fillings are more resistant and more durable than others. I, with Miller, ascribe it, in part at least, to this fact, though something may be due to the greater care with which fillings of the precious metals are made. Gold plates are better borne by the oral mucous membrane than those made of hard rubber. It seems to be proved that various metals, more especially mercury, silver, and gold, have antiseptic properties; whilst zinc, lead, and iron seem to be quite powerless in this regard. The laboratory experiments and clinical researches of Credé and Beyer were so entirely satisfactory that I resolved to try the new antiseptics in dental work. In the sterilization of root sinuses I thought that they would be specially useful. My trials were made upon teeth with freshly killed pulps, as well as upon those in which the pulps had become gangrenous, and include about one hundred cases. And I may state at once that my hopes were not disappointed. The results that I have obtained during the last half-year have been entirely satisfactory to me, and I can recommend these preparations to my colleagues in the very warmest manner.

I directed all the patients that I treated with the silver salts to return to me at once as soon as any pain occurred in the teeth that had been treated. Up to the time of this present writing only three have reappeared with periostitis; and in two out of these three the defects in the molars were distal, and difficult to get at.

My method of root-treatment is the following: I open the pulp-cavity with a suitable trephine and, if the pulp is gangrenous, clean it out as thoroughly as possible with a thin probe. Then I thoroughly and repeatedly inject out the root-canal with a freshly prepared and dilute solution (1 to 2000) of the lactate of silver. Then I apply the rubber dam and dry the cavity, first with cotton, and then with the warm-air injector, to the completeness of which procedure I attach the greatest weight. For I believe that even if dead nerve-tissue remains behind, further decomposition and the development of the gases of putrefaction cannot so readily occur if all moisture is thoroughly removed, and the mummification of whatever of nerve-tissue is left behind is effected. Here the powdered citrate of silver is of especial value, since it not only permanently disinfects the decomposition products that remain, but acts as a desiccant also in consequence of its powdered form. As is well known, the difficulty in the sterilization of the root-canals depends on the difficulty of thoroughly applying the antiseptic to it. I believe that it is best done by applying the citrate through an insufflator, to the nozzle of which a rubber tube with a very small orifice is attached; this permits the application to be made to all the sinuosities or even the distal canal.

The pulverization is fairly forcible, and, if the lumen of the canal is sufficiently large, I do not doubt that the particles of the drug reach the very ends of the sinuses. If the insufflator does not seem to have effected this, I apply the powdered citrate to the depths of the canal on a thin probe.

Recently devitalized pulps I usually fill at one sitting, simply dusting in the citrate after opening the cavity with a sterilized bur, and filling with tin or gutta-percha in the usual manner. When the root-canals are putrid, I deem it necessary to make two or three applications and insufflations of the citrate or the lactate before proceeding to the permanent filling. It is surprising to see, in most cases, that after the first introduction of the silver salt the odor of decomposition entirely disappears. I have seen no discoloration of the teeth in the cases that have returned to me; it is true, however, that they were all molar cases. Only the cavities were colored black. Irritation symptoms after the applications were never noted.

I do not claim that this method of the treatment of roots is the only proper one; for there are almost as many different methods as there are practitioners of dentistry. But with these antiseptics I have formulated a suitable and effective system of treatment.

In conclusion, let me state that I have used the silver preparations in various other diseases. I have used the gray silver gauze in one case of empyema of the antrum of Highmore, and as a tampon for the hemorrhage following extractions, and also the dilute solutions as gargles in stomatitis, and I have obtained like satisfactory results with them.

The comparatively small number of cases over which my experience extends are too few upon which to base a final judgment. But the good results certainly enable me to recommend that these silver salts be extensively experimented with and tried by others, and I should be glad to stimulate those of my colleagues who have not yet used these antiseptics to do so.

